# Interactions between mosquito genetic background and *Wolbachia* strain affect dengue virus blocking and fitness in South American populations of *Aedes aegypti*

**DOI:** 10.1371/journal.pntd.0014403

**Published:** 2026-05-27

**Authors:** Suk Lan Ser, Nina L. Dennington, Heather I. Engler, Makael L. Harris, Matthew J. Jones, Frank W. Avila, Eric P. Caragata, Ary A. Hoffmann, Rafael Maciel de Freitas, Elizabeth A. McGraw

**Affiliations:** 1 Department of Biology, The Pennsylvania State University, University Park, Pennsylvania, United States of America; 2 The Huck Institutes of the Life Sciences, The Pennsylvania State University, University Park, Pennsylvania, United States of America; 3 Max Planck Tandem Group in Mosquito Reproductive Biology, Universidad de Antioquia, Medellín, Antioquia, Colombia; 4 Florida Medical Entomology Laboratory, Department of Entomology and Nematology, Institute of Food and Agricultural Sciences, University of Florida, Vero Beach, Florida, United States of America; 5 Pest and Environmental Research Group, Bio21 Institute, University of Melbourne, Melbourne, Victoria, Australia; 6 Department of Arbovirology, Bernhard Nocht Institute for Tropical Medicine, Hamburg, Germany; 7 Laboratório de Mosquitos Transmissores de Hematozoários, Instituto Oswaldo Cruz, Fiocruz, Rio de Janeiro, Brazil; University of Queensland, AUSTRALIA

## Abstract

The mosquito *Aedes aegypti* is the primary vector of dengue virus, and the release of mosquitoes infected with the bacterium *Wolbachia* is an increasingly used strategy to reduce dengue transmission. This approach relies on *Wolbachia*’s ability to suppress virus replication within the mosquito, but the effectiveness of virus suppression and the associated fitness costs may depend on interactions among mosquito genetic background, *Wolbachia* strain, and dengue virus type. Here, we examined these interactions using mosquito populations from Brazil, Paraguay, and Peru infected with two *Wolbachia* strains, *w*MelM and *w*AlbB, and exposed to dengue virus serotype 1 or serotype 2. We measured dengue virus infection in transmission-relevant tissues and quantified mosquito survival, development, and reproduction under controlled laboratory conditions. *Wolbachia* infection reduced dengue virus infection overall, but the strength of virus suppression varied among mosquito populations, with the strongest blocking observed in Brazilian mosquitoes and the weakest in Peruvian mosquitoes. Across all populations, the *w*MelM strain consistently produced stronger dengue virus suppression than *w*AlbB, whereas mosquitoes infected with *w*AlbB generally exhibited higher fitness, with fitness effects varying among mosquito populations and life-history traits. Together, these results show that genetic interactions among the mosquito, *Wolbachia*, and dengue virus shape both virus blocking and mosquito fitness, highlighting trade-offs that may influence the establishment and long-term effectiveness of *Wolbachia*-based dengue control programs.

## Author summary

Dengue virus is transmitted by the mosquito *Aedes aegypti*, causing large outbreaks in many parts of the world. One promising way to reduce dengue virus transmission is to release mosquitoes infected with a naturally occurring bacterium called *Wolbachia*, which interferes with the virus inside the mosquito. However, this strategy has not worked equally well in all locations, and the reasons for this variability are not fully understood. In this study, we tested whether differences in mosquito populations, *Wolbachia* strains, and dengue virus types influence how well this approach works. We compared mosquitoes from Brazil, Paraguay, and Peru infected with two commonly used *Wolbachia* strains and exposed them to two dengue virus types. We found that one *Wolbachia* strain was better at suppressing dengue virus, while the other allowed mosquitoes to survive and reproduce more effectively. Importantly, these effects depended strongly on the mosquito population being tested, with mosquitoes from Brazil showing the strongest virus suppression and those from Peru showing the weakest. Our results show that there is no single *Wolbachia* strain that performs best in the settings we studied and that matching *Wolbachia* strains to local mosquito populations may be important for improving the long-term success of dengue control programs.

## Introduction

Dengue fever has become an escalating public health crisis across the Americas, with cases surging to record levels. In 2024, over 14 million cases were reported in the region [1]. Notably, dengue is now being reported in new areas, suggesting a geographic expansion of virus transmission driven by changing environmental conditions and increased habitat suitability for the mosquito vectors. The virus, now endemic in most Latin American and Caribbean countries, is posing a persistent threat to regional health systems. Limited antiviral treatments and restricted access to effective vaccines have left vector control as the primary strategy for curbing dengue virus (DENV) transmission [2]. However, traditional methods such as insecticide spraying and larval habitat reduction have shown declining effectiveness due to the evolution of insecticide resistance [3] in *Aedes aegypti* (*Ae. aegypti*) populations and inconsistent implementation of these methods, often hindered by social and logistical challenges that allow mosquito densities to rebound [4]. These challenges underline the need for innovative, resilient vector control strategies that work under real-world ecological and operational conditions. The release of the insect endosymbiont, *Wolbachia*, into mosquito populations is one such promising strategy [5].

In one approach using *Wolbachia*, the microbe is released into mosquito populations where it spreads, ‘replacing’ the local *Wolbachia*-free population. *Wolbachia* is maternally transmitted and spreads through mosquito populations via cytoplasmic incompatibility, an effect that gives infected females a reproductive advantage [6]. Crucially, *Wolbachia* also inhibits arbovirus replication within the mosquito host, thereby reducing the transmission potential of coinfecting RNA viruses, including dengue, Zika, chikungunya, and Yellow Fever, once *Wolbachia* has established [7]. This ‘pathogen blocking’ trait is believed to arise through multiple mechanisms, including immune activation [8], oxidative stress [9], and disruptions to cellular processes such as lipid transport [10], cell adhesion [11], and amino acid availability [12] in the vector. These physiological changes collectively create a hostile intracellular environment that impairs viral replication and dissemination to transmission-relevant tissues, including, the salivary glands.

Although field deployments of *Wolbachia*-infected mosquitoes have been encouraging—both in terms of *Wolbachia* establishment and subsequent reductions in dengue transmission—these outcomes have depended on the site where releases have taken place. In Yogyakarta, Indonesia [13], and North Queensland, Australia [14], large-scale releases have led to reductions in dengue incidence ranging from 77% to 99% in targeted regions. However, outcomes have been less consistent in other locations. In Rio de Janeiro, Brazil [15,16], and Vietnam [17], and more recently Medellín, Colombia [18], *Wolbachia* establishment has been variable or declined following release cessation, with recent surveys reporting low and highly heterogenous prevalence (~32% or less) in local *Ae. aegypti* populations. More recently, a field trial of *w*AlbB-infected mosquitoes in Malaysia reported a 62% reduction in dengue cases, highlighting promising but variable success [19]. *Wolbachia* strain *w*AlbB, originally derived from *Aedes albopictus*, is known for its relatively high thermal stability and ability to persist under field-relevant temperature conditions, though its pathogen-blocking efficacy can be more variable [20]. In contrast, *w*MelM, a variant of the *w*Mel strain originating from *Drosophila melanogaster*, has been associated with strong dengue virus blocking, and improved thermal tolerance compared to the original *w*Mel strain, although environmental conditions can still influence its performance [7,21]. These differences in release outcomes across geographic locations suggest that *Wolbachia*-based strategies might depend on a combination of genetic, ecological, and operational factors that remain insufficiently understood.

Environmental factors, such as temperature and humidity, can influence *Wolbachia*’s density within mosquitoes, which in turn impacts both pathogen-blocking efficiency and invasion potential [22,23]. In addition to these abiotic factors, the genetic backgrounds of both the mosquito and the *Wolbachia* strain have been shown to modulate the strength of virus blocking. For instance, in one study comparing *Ae. aegypti* populations from Mexico and Singapore infected with the same *w*AlbB strain, there were differences in the strength of DENV blocking, suggesting a role for mosquito genotype [24]. In another study with an Australian *Ae. aegypti* population infected with either *w*MelM or *w*AlbB, differences in DENV susceptibility were detected, indicating a role for *Wolbachia* genotype [25]. Finally, in a study of a single genetic background with one strain of *Wolbachia,* there was variation in blocking efficacy across different DENV serotypes (genetically and antigenically distinct variants of the virus), indicating the importance of virus genotype [26]. Importantly, *Wolbachia* infection can also reduce mosquito fitness, via such measured traits as fecundity [27], lifespan [28] and fertility [29], affecting its ability to invade and persist in the field. These fitness effects also vary by host and symbiont genotype [30]. This body of work highlights the ability for mosquito:virus:*Wolbachia* genotypes to make or break the efficacy of the biocontrol agent, which in turn also depends on environmental conditions.

Given the rising urgency of dengue control in the Americas and the scale-up of *Wolbachia* release programs across Brazil, Colombia, Honduras, and Puerto Rico, there is a clear need to understand how local genetic variation might influence intervention success and possibly explain past failures. This study uses a genotype-by-genotype-by-genotype (G × G × G) framework to evaluate how mosquito genetic background, *Wolbachia* strain, and DENV serotype interact to shape virus blocking and mosquito fitness- factors that will either promote or limit the success of *Wolbachia*-based interventions. We have selected mosquito populations from across Brazil, Paraguay, and Peru, the two leading *Wolbachia* release strains (*w*MelM and *w*AlbB), and two of the commonly circulating DENV serotypes that are also highly divergent from one another genetically [26,31]. This work provides a basis for understanding how interacting genetic factors influence both the efficacy and sustainability of *Wolbachia*-based dengue control, offering insights that can help refine and localize intervention strategies across the globe.

## Methods

### Mosquitoes and colony maintenance

*Ae. aegypti* mosquitoes were collected from field sites across three South American countries: Brazil (Rio de Janeiro), Paraguay (Ciudad del Este), and Peru (Campo Verde). Eggs were collected using 60 ovitraps homogeneously spread in dengue endemic neighborhoods of each location, and paddles were replaced weekly until obtaining a minimum of 10,000 eggs per site to capture local genetic diversity*.* The wild mosquito populations were screened for *Wolbachia* infection using diagnostic PCR assays targeting *Wolbachia*-specific genes to confirm the absence of infection, following established protocols [30,32]. Only *Wolbachia*-free populations were used to establish experimental lines. The *w*MelM donor line was obtained from Ary Hoffmann (University of Melbourne) [33], and the *w*AlbB donor line was obtained from Zhiyong Xi (Michigan State University), that had previously been established in *Aedes aegypti* through backcrossing into a *Wolbachia*-free line from Mérida, Mexico [24].*Wolbachia* strains, *w*MelM [34], and *w*AlbB [33], were first introgressed into each country line via repeated backcrossing using infected donor lines. For each population, infected females were backcrossed to males from the corresponding *Wolbachia*-free country line for six consecutive generations. This approach preserves the maternally inherited *Wolbachia* infection while progressively replacing the nuclear genetic background of the donor line. After six generations, the nuclear genome (>98%) is expected to reflect the recipient population, minimizing donor genetic carryover while maintaining colony viability. Both *w*MelM and *w*AlbB strains were originally transinfected into *Ae. aegypti* in previous studies [24,33] and have since been maintained in laboratory colonies for multiple generations prior to their use in this study. Following backcrossing, qPCR assays targeting strain-specific markers were performed to confirm the presence and identity of the respective *Wolbachia* strain in each experimental population [30,32]. A *Wolbachia*-free strain was maintained as a control for each population, resulting in nine experimental populations. All mosquito lines were reared under standard laboratory conditions: 26°C, 75% relative humidity, and a 12-hour light/dark photoperiod. Larvae were provided fish food (TetraMin) *ad libitum* throughout their development. Adult mosquitoes were maintained on 10% sucrose and were not blood-fed except where explicitly stated for experimental assays.

### Vector competence assay

The DENV serotypes/strains used for this experiment were DENV-1 strain FR-50 (GenBank accession number FJ432734.1) and DENV-2 strain ET-300 (GenBank accession number EF440433.1). The viruses were cultured in *Ae. albopictus* C6/36 cells (Sigma), following previously established protocols [35]. C6/36 cells were maintained in RPMI 1640 medium (Life Technologies) supplemented with 10% heat-inactivated fetal bovine serum (FBS), 20mM HEPES buffer (Sigma-Aldrich), and 1% penicillin-streptomycin (Life Technologies). Cells were grown to 80% confluence in T75 flasks before inoculation with DENV-1 or DENV-2. Infected cultures were incubated at 27°C for 7 days, and the supernatant was harvested and titrated to a final viral load (as per below) of 10^7^ DENV copies per ml. The supernatant was mixed in a 1:1 ratio with blood from anonymous human donors (BioIVT) before feeding. Donor blood was not screened for preexisting immunity to DENV or other flaviviruses. Adult female mosquitoes at 7 days post-eclosion were deprived of sucrose for 24 hours before the infectious feed. The mosquitoes were fed using a Hemotek artificial feeder (Hemotek Ltd., UK) at 37 ^∘^C using pig intestine (sausage casing) as the membrane. The final virus concentration in the blood was 1.20e^7^ DENV copies/ml for DENV-1 and 2.40e^7^ DENV copies/ml for DENV-2. After feeding, mosquitoes were anesthetized on ice, and only the fully engorged were selected for subsequent experiments. Engorged mosquitoes were then placed into 32 oz paper soup cups with mesh lids, with each cup containing 12 individuals. A total of 54 groups were established, with each treatment represented by a single cup; all groups were set up simultaneously as part of a single experimental infection assay. All groups were provided with 10% sucrose-soaked cotton balls, which were refreshed daily. All mosquitoes were maintained under the same environmental conditions as the stock lines. At 5, 10, and 15 days post-infection, mosquitoes were anesthetized on ice, and specific tissues (salivary gland and carcass) were dissected under a microscope. Dissected tissues and body parts were stored separately in 1.5 ml microcentrifuge tubes (Sarstedt, Nümbrecht, Germany) containing 300μl of DNA/RNA shield and a 2.8mm ceramic bead. Samples were homogenized using a Bead Ruptor Elite (Omni International, USA) and kept frozen at -80°C until further processing.

### Mosquito nucleic acid extraction

Total RNA was extracted using the Zymo Quick-DNA/RNA Pathogen MagBead Kit (Zymo Research, Cat.No R2146) according to the manufacturer’s protocol. The extraction was performed using a MagMax Express 96 (Applied Biosystems), and DNA/RNA was eluted in 50μl ZymoBIOMICS DNAase/RNase-free water at room temperature. RNA was eluted in 50μl RNase-free water and then treated with 5 units of DNase I (Sigma-Aldrich) at room temperature for 15 mins, followed by inactivation with 50mM EDTA at 70°C for 10 mins. RNA and DNA extractions were performed using the column-based Direct-zol DNA/RNA miniprep kit. RNA was eluted into 50μl RNase-free water, followed by DNA elution in 50μl Direct-zol DNA elution buffer. All RNA quantification assays used the same endogenous control (18S ribosomal RNA) across all mosquito populations and treatments to ensure consistency in normalization.

### Dengue virus quantification

DENV was quantified using the TaqMan Fast Virus 1-step Master Mix (Thermo Fisher Scientific) in 10 µl reaction volumes, with DENV-2 specific primers and probes for qRT-PCR, following previously described methods [11]. The protocol consisted of a reverse transcription step at 50°C for 5 minutes, an initial denaturation at 95°C for 20 seconds, and amplification cycles of 95°C for 3 seconds followed by 60°C for 30 seconds. Absolute quantification was achieved using a standard curve generated from a known concentration of a DENV-2 genomic fragment. This fragment was cloned into a plasmid, transformed into *Escherichia coli*, and prepared as described previously [11]. The linearized and purified fragment was serially diluted from 10^6^ to 10^2^ copies to create a standard curve for DNA amplification. Each 96-well plate included the standard curve and negative controls, both run as duplicates.

### Survival and fecundity

Female adult mosquitoes from the nine experimental populations were maintained under standard conditions in 5 groups of 20 individuals each per cup. Survival was monitored daily until all individuals died. Mosquitoes in the survival assay were maintained with cotton balls soaked in 10% sucrose solution, which were replaced daily, and were never blood fed during the experiment. For fecundity, adult female mosquitoes 7 days post-eclosion (after mating) were sorted by experimental populations and divided into groups. Each population group consisted of 30 females housed in three BugDorm-4S1515 (5.4-liter) insect-rearing cages (10 individuals per cage). Each cage had a small urine specimen cup (180ml) lined with filter paper and filled with deionized water to keep the paper moist. Throughout the assay, mosquitoes had access to 10% sucrose water except for 24 hours prior to each blood meal consisting of human blood. At 7 days post-eclosion, females were deprived of sucrose for 24 hours before receiving a noninfectious blood meal, as above. Females were allowed to oviposit over three days per cycle, during which the filter paper was replaced every three days. This process was repeated at 14 and 21 days post-eclosion to capture the second and third gonotrophic cycles, respectively, and eggs were manually counted each time. After each blood meal, the number of surviving females was recorded, and the average fecundity for each gonotrophic cycle was calculated by dividing the total number of eggs by the number of live females per replicate and experimental population.

### Larval development

Eggs from each of the nine experimental populations were hatched under standard laboratory conditions as described above. After 24 hours, 150 first-instar larvae from each population were randomly selected and divided into five 250 ml plastic cups, with 30 larvae per cup. Each cup contained deionized water and was provided with fish food (TetraMin) *ad libitum.* Larvae were continuously fed until the onset of pupation. Once pupation began, the amount of food provided was adjusted according to the remaining larvae in each cup to prevent overfeeding. Pupae, both live and dead, were removed daily, counted, and placed in individual 30 ml plastic cups containing water from their original larval environment to facilitate eclosion. These cups were added to respective BugDorm-4S1515 (5.4-liter) insect-rearing cages (Megaview Science Co., Ltd., Taiwan) with continuous access to 10% sucrose solution. The number of adults emerging each day was recorded to assess development time.

### Model development for the prediction of mosquito fitness (*r*) fitness

To calculate population-level fitness, we used a stage-structured matrix projection model, which we parameterized from our data as previously described [11,36,37]. We describe the change in population over time:

𝑁𝑡+1 = 𝑀𝑁𝑡

N is the abundance in the stage at a given time (t), and M is the population projection matrix. Fecundity is the first matrix row, populated by fecundity or the average number of offspring produced per female at a given age, of which we included two gonotrophic cycles. The sub-diagonal of the matrix consists of the survival probability from a given age to age i + 1. The stage-structured population dynamics are the product of the transition matrix and the stage-structured population size vector across time. The stable stage distribution of the abundance vector is reached after repeated multiplication, and the dominant eigenvalue of the system is the finite population rate of increase (𝜆). The intrinsic rate of population growth is

𝑟_𝑚𝑎𝑥_ = log(𝜆)

This is the population fitness representing the population’s ability to reproduce, and as such, negative r_max_ indicates a population that is in decline, while positive r_max_ indicates population growth.

For each trait, replicate values were resampled with replacement with 10,000 iterations per treatment to account for variation in the number of replicates between trait measurements. Note that we maintained the order of survival probability across time for resampling in adult survival. We incorporated our resampled iterations for each trait with a Bayesian inference framework to estimate the posterior distribution of r_max_ for each treatment. The model assumed that each estimate of r_max_ has a normal distribution with a mean of 𝜇_𝑟𝑚𝑎𝑥_ and a precision parameter 𝜏_𝑟𝑚𝑎𝑥_ where 𝜏 is the inverse of the variance. We assigned weekly informative priors with a mean (𝜇) of 0, a precision (𝜏) of 0.001, and a uniform prior between 0 and 10 for the standard deviation (𝜎_𝑟𝑚𝑎𝑥_) allowing our data to inform the posterior distribution.

We fit the models using a Markov Chain Monte Carlo (MCMC) sampling as implemented in JAGS, using the *R2jags* along with the *popbio* package [38–40]. For each treatment group we ran three MCMC chains with 10,000 iterations and a 3,000 step burn-in. We used these samples for the posterior distribution. We then summarized the r_max_ by calculating the mean and 95% highest posterior density (HPD) interval. The HPD includes the smallest continuous range, containing 95% of the probability, which is the credible interval implemented by the *coda* package [41]. We then used pairwise comparisons of the posterior difference of samples to understand the probability that one treatment’s r_max_ was more significant than another.

### Parameterizing of life-history traits

Individuals’ survival and life stage duration (larva to pupa to adult stages) were measured through pupation and adult emergence time. The first instar larval emergence was assumed to be one day. Adult survival elements were populated using a continuous survival proportion from a Kaplan-Meier survival function in the R survival package *survival* and *survminer* [42]. Fecundity measurements were the average eggs per female per gonotrophic cycle with two stages for the first two gonotrophic cycles. The assumption of unlimited resources, density, and constant laboratory conditions constrains these estimates.

### Statistical analysis

All statistical analyses were performed using R (version 4.3.2) or JMP Pro (version 18.1.0, SAS Institute Inc., Cary, NC, 1989–2025), depending on the assay. For the vector competence assay (dengue prevalence), analyses were conducted in R. A chi-squared binomial generalized linear model (GLM) with a logit link was used to compare infection prevalence among treatments within specific mosquito tissues. Infection prevalence was analyzed as the number infected over the total number of individuals, with ‘Dengue serotype,’ ‘Mosquito strain,’ ‘*Wolbachia* strain,’ and ‘Days Post-Infection (DPI)’ included as categorical fixed effects. *Wolbachia* infection status (*w*AlbB, *w*MelM, and *Wolbachia*-free) was modeled as a single factor, with comparisons to the *Wolbachia*-free group representing overall blocking effects, and direct comparisons between *w*AlbB and *w*MelM reflecting strain-specific differences. Differences between models including or excluding interactions were assessed using ANOVA. For the survival assay, Kaplan-Meier survival curves were generated in R, stratified by *Wolbachia* strain, to visualize survival differences. Pairwise comparisons were conducted using the log-rank test to identify significant differences between populations or treatments. For the fecundity assay, ANOVA was performed in R to assess the effects of mosquito population (country), *Wolbachia* infection status, number of blood meals (gonotrophic cycle), and their interactions on egg production. For the larval development assay, ANOVA was performed to assess the effects of mosquito population (country), *Wolbachia* infection status, and their interaction on development time. All factors were treated as fixed effects, and pairwise comparisons were conducted using Tukey’s post hoc tests where appropriate. The statistical analysis of dengue viral loads in mosquitoes from the vector competence assay was conducted in JMP Pro. Mixed-effects models were fit by maximum likelihood and statistically compared using Tukey’s post-hoc tests to evaluate interactions among population and treatment groups.

## Results

### *w*MelM Exhibits stronger DENV blocking than *w*AlbB in South American *Ae. aegypti*

**Fig 1:**
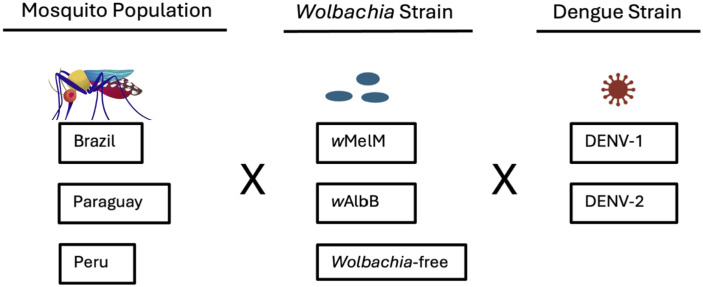
Schematic representation of the GxGxG experimental design investigating *Wolbachia*-mediated pathogen blocking in mosquitoes from Brazil, Paraguay, and Peru. Three *Wolbachia* conditions were tested: *w*AlbB, *w*MelM, and *Wolbachia*-free controls. Each *Wolbachia* strain was backcrossed into *Ae. aegypti* populations from each country-based population and then exposed to either dengue virus serotype 1 (DENV-1) or dengue virus serotype 2 (DENV-2). Viral infection was assessed at 5,10, and 15 days post-infection (dpi) in two tissues: salivary glands and carcass.

To investigate the effects of *Wolbachia* strain and mosquito genetic background on DENV blocking, we generated multiple experimental populations through backcrossing. Specifically, we introduced *w*AlbB and *w*MelM strains into *Ae. aegypti* populations from Brazil, Paraguay, and Peru. The *Wolbachia*-infected and wildtype pair-matched control lines were then fed dengue virus serotype 1 (DENV-1) or serotype 2 (DENV-2) to assess the impact on *Wolbachia-*mediated pathogen blocking (Fig 1).

We assessed both DENV prevalence and viral load in several tissues for the different mosquito populations at three time points post-infection. We hypothesized that mosquito genetic background, *Wolbachia* strain, and DENV strain would influence the extent of *Wolbachia*-mediated pathogen blocking. For both prevalence and load, the three main effects of population, *Wolbachia status*, and DENV serotype were all significant, (S1–S3 Tables). The three-way interactions for prevalence (*χ*^*2*^ = 594.0, df = 4, p < 0.0001) and viral load (F(4,1188) =7.39, p < 0.0001), were also significant, revealing the presence of GxGxG effects.

**Fig 2:**
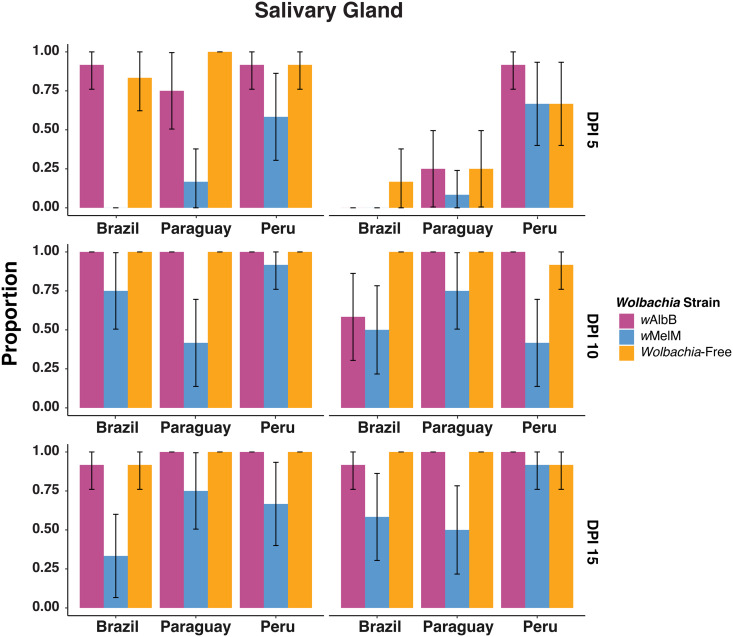
Dengue virus prevalence in the salivary glands of mosquito populations from Brazil, Paraguay, and Peru tested with *Wolbachia* strains *w*AlbB, *w*MelM, and *Wolbachia* -free controls. Each population was exposed to **a)** DENV-1 or **b)** DENV-2, and mosquitoes were subsequently collected at 5-, 10-, and 15-days post-infection (DPI) to assess dengue prevalence in the salivary glands. Bars represent the proportion of infected mosquitoes per treatment group (n = 12 individuals per treatment group at each time point), with error bars indicating standard error.

**Fig 3:**
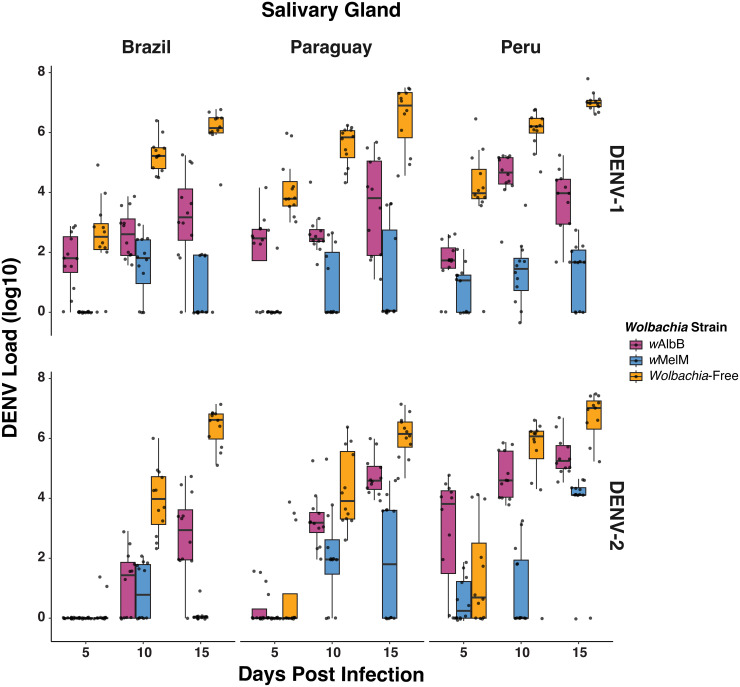
Dengue viral load in the salivary glands of mosquito populations from Brazil, Paraguay, and Peru tested with *Wolbachia* strains *w*AlbB, *w*MelM, and *Wolbachia* -free controls. Each population was exposed to DENV-1 or DENV-2, and dengue viral loads were assessed in salivary glands collected at 5-, 10-, and 15- days post-infection (DPI). Bars represent median viral load and whiskers indicating the 95% confidence intervals. Each dot represents salivary gland tissue dissected from a single individual. n = 12 individuals per treatment group at each time point.

Our results show that *Wolbachia* infection significantly reduced both DENV prevalence and viral load, with *w*MelM-infected mosquitoes exhibiting the strongest blocking effect, regardless of mosquito population. Specifically, *w*MelM mosquitoes had lower DENV prevalence in the salivary glands (Fig 2: Generalized Linear Model: “*Wolbachia*”: *χ*^*2*^ = 87.51, df = 2, p < 0.0001) compared to *w*AlbB-infected and *Wolbachia*-free controls. This pattern was mirrored in viral load, where *w*MelM-infected mosquitoes had significantly lower DENV loads in the salivary glands (Fig 3: Mixed-Effects Model: *“Wolbachia”:* F = 697.62*,* df = 2*,* p < 0.0001). A similar trend was observed in the carcass for both DENV prevalence and viral load (S1 and S2 Fig), reinforcing the robust blocking effect of *w*MelM across tissues. Additionally, *Wolbachia* infection was more effective at blocking DENV-2 than DENV-1, as measured by prevalence (“DENV serotype”: *χ*^*2*^ = 25.01, df = 1, p < 0.0001*)* and viral load (“DENV serotype”: F(1,1188) =27.73, p < 0.0001) across mosquito populations. Across both *Wolbachia* strains (*w*MelM and *w*AlbB), the strength of *Wolbachia*-mediated blocking varied by country-based population, with mosquitoes from the Brazil line frequently exhibiting stronger blocking and Peruvian lines comparatively weaker blocking across strains, serotypes, and tissues. This population-level trend was consistent across both salivary glands (Figs 2–3) and carcasses (S1 and S2 Figs) for both DENV prevalence and viral load. Full GLM results are provided in S1 and S2 Tables. Detailed pairwise comparisons are provided in S1 Data.

### Mosquito fecundity varies independently of *Wolbachia* strain and genetic background

**Fig 4:**
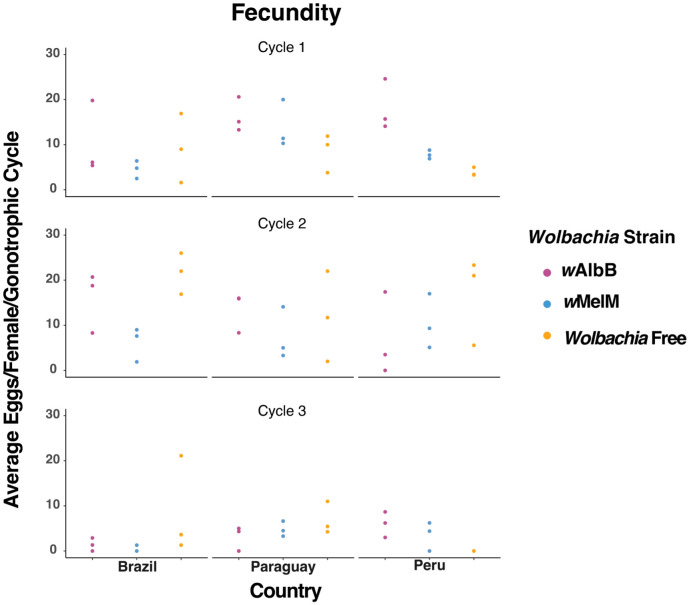
Fecundity of mosquito populations from Brazil, Paraguay, and Peru tested with *Wolbachia* strains *w*AlbB, *w*MelM, and *Wolbachia*-free controls. The average number of eggs per female was recorded over three gonotrophic cycles to assess reproductive output across different *Wolbachia* strains and geographic backgrounds. Data are presented as a dot plot, with each point representing a biological replicate (one cage of 10 females). Egg counts were pooled within each replicate and normalized by the number of surviving females. Each treatment group consisted of three replicate cages (n = 3).

We then evaluated the effect of *Wolbachia* strain and the mosquito’s genetic background on *Ae. aegypti* fecundity measured over three gonotrophic cycles. There was no significant interaction between *Wolbachia* strain and mosquito population, nor between gonotrophic cycle and either factor, indicating independent effects on fecundity. Our analysis revealed that only gonotrophic cycle was significant (ANOVA: F(1,63) = 12.55*,* p = 0.00075), while neither *Wolbachia* strain (F(2,63) = 2.33*,* p = 0.105) nor mosquito population (F(2,63) = 0.321, p = 0.726) had an effect. As shown in Fig 4, fecundity declined across gonotrophic cycles, with the number of eggs per female generally decreasing from the first to the third cycle. However, there was no clear trend associated with *Wolbachia* infection or geographic background, indicating that reproductive output is primarily influenced by the gonotrophic cycle rather than *Wolbachia* presence or country of origin (see S3 Table for full ANOVA analysis), with no evidence of improved fecundity in either *w*MelM or *w*AlbB-infected mosquitoes relative to wildtype.

### *Wolbachia* reduces *Ae. aegypti* survival, with strongest effects in Brazil

**Fig 5:**
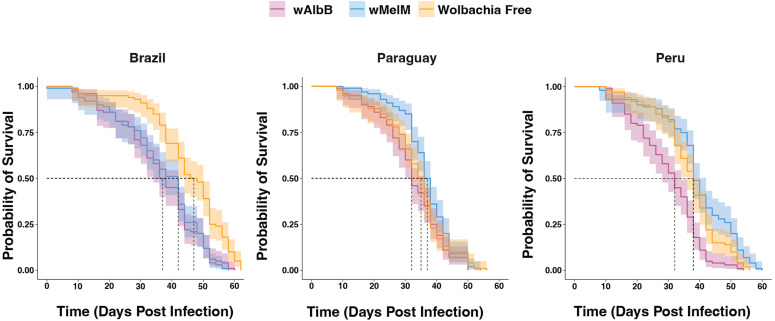
Effect of *Wolbachia* infection on the relative fitness of mosquito populations from Brazil, Paraguay, and Peru. The colored lines represent the probability of survival for 100 individual mosquitoes per treatment group (five independent biological replicates of 20 mosquitoes each), with shaded areas representing 95% confidence intervals. The dotted line indicates the time point at which 50% of the individuals in each group survived.

To analyze the effect of *Wolbachia* infection on mosquito survival, we performed survival analysis using a pairwise log-rank test across the different South American mosquito populations in the absence of DENV infection. Among *Wolbachia*-free control mosquitoes, there were significant differences in baseline survival between populations, with the Brazilian line exhibiting the highest survival, followed by Peru, and then Paraguay (Fig 5). Overall, our results show that *Wolbachia* infection significantly reduced overall survival in most populations, but the magnitude of these effects varied across populations. The greatest reduction was observed in the Brazilian population, where *Wolbachia*-infected mosquitoes exhibited the shortest lifespan, reaching 50% death sooner than their uninfected counterparts, with median survival reduced by approximately 1.25-1.3-fold. This trend was consistent across both *w*MelM and *w*AlbB strains (log-rank; *χ*^*2*^ = 20.31, df = 1, p < 0.05). While both *Wolbachia* strains reduced the survival compared to *Wolbachia*-free controls, differences between *w*MelM and *w*AlbB were dependent on the mosquito population. In Brazil, there was no significant difference in survival between mosquitoes infected with *w*MelM and those infected with *w*AlbB (log-rank; *χ*^*2*^ = 0.00015, df = 1, p = 0.99). In contrast, in Paraguay, *w*MelM-infected mosquitoes tended to have a slightly longer lifespan than those infected with *w*AlbB (log-rank; *χ*^*2*^ = 7.51, df = 1, p < 0.05). A similar trend was also observed in Peru, where *w*AlbB-infected mosquitoes had a shorter lifespan compared to those infected with *w*MelM (log-rank; *χ*^*2*^ = 38.98, df = 1, p < 0.05) (see S4 Table for details on individual pairwise comparisons).

### Effect of *Wolbachia* infection on mosquito development rate depends on mosquito’s genetic background

**Fig 6:**
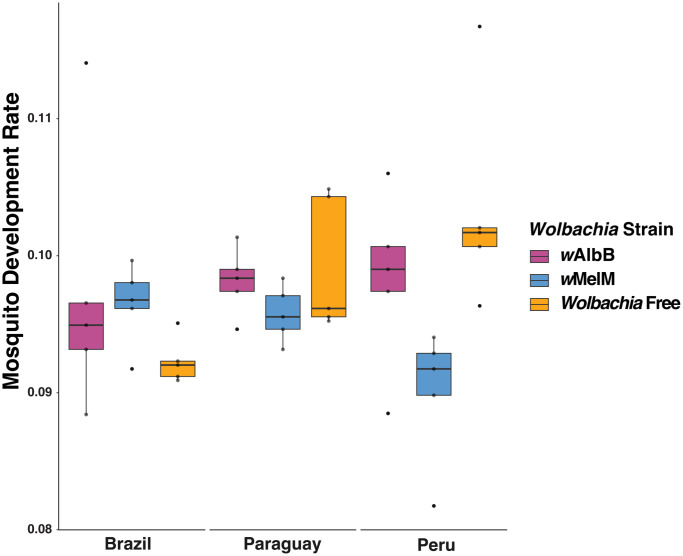
Mosquito development rate of populations from Brazil, Paraguay, and Peru tested with *Wolbachia* strains *w*AlbB, *w*MelM, and *Wolbachia*-free controls. The development rate, calculated as the inverse of the time taken (days) for first-instar larvae to reach the adult stage, is presented for each group. Data are displayed as boxplots, with bars representing median development rate and whiskers indicating the 95% confidence intervals. Each dot represents a replicate group. The sample size is n = 5 replicates per treatment, with 30 individual larvae included at the start of each experiment.

We examined mosquito development rate, calculated as the inverse of the time (in days) it took for mosquitoes to develop from the first instar larval stage to the adult stage across different South American mosquito populations. Here, we show that neither *Wolbachia* status (ANOVA: F(2,36) = 2.80*,* p = 0.074) nor the mosquito’s genetic background (F(2,36) = 0.77*,* p = 0.47) alone significantly influenced mosquito development rate. However, there was a significant interaction between *Wolbachia* status and population (F(4,36) = 3.38, p = 0.019), indicating that the effect of *Wolbachia* infection on development rate depends on the mosquito’s genetic background. As shown in Fig 6, development rate varied inconsistently across populations and *Wolbachia* strains. In the Brazil line, mosquitoes infected with *w*AlbB and *w*MelM exhibited slightly higher development rates compared to *Wolbachia*-free controls. In the Paraguay line, development rates were more consistent across all *Wolbachia* statuses, with no clear pattern of suppression or enhancement. In Peru, however, *w*MelM-infected mosquitoes tended to have slower development rates than *Wolbachia*-free controls. This variation suggests that the influence of *Wolbachia* on mosquito development rate is background-dependent.

### *w*AlbB-infected mosquitoes exhibit higher population growth rates across genetic backgrounds

**Fig 7:**
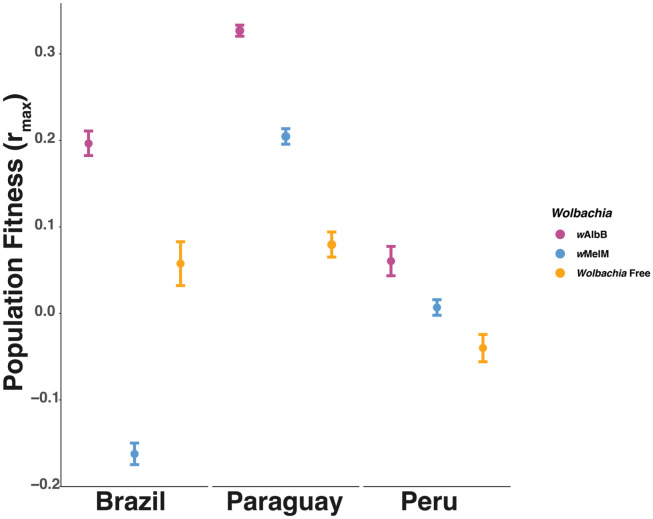
Fitness (r_max_) derived from life-history traits measured in mosquitoes reared from three populations (Brazil, Paraguay, Peru) and three *Wolbachia* infection statuses (*w*AlbB, *w*MelM, and *Wolbachia* -free controls). The point indicates the mean model fit, and the error bars indicate the 95% credible intervals.

We compiled these life-history traits into a composite fitness model to assess how the interaction between mosquito genetic background and *Wolbachia* infection influences population growth rate (*r*_max_). Our results show a consistent pattern across populations: mosquitoes infected with *w*AlbB exhibited the highest population growth (Fig 7). In Brazil, *w*AlbB-infected mosquitoes had a 2.3-fold higher *r*_max_ compared to *w*MelM-infected mosquitoes. In Peru and Paraguay, the difference was smaller but still notable—approximately 1.8-fold and 1.5-fold, respectively. Although some similarities were observed between populations, treatment groups were significantly different from each other (posterior probability, Δ > 0; see S5 Table). It is also important to note that in this model, not all life-history traits contribute equally to population fitness. For example, depending on the dataset, survival may have a stronger influence on *r*_max_ than development rate or fecundity. To explore this further, we examined the elasticity of each trait to determine how much each one contributed to overall fitness; details of the elasticity analysis are provided in the supplementary materials. Fecundity juvenile survival showed the highest elasticity, indicating that small changes in these traits would have the greatest impact on *r*_max_. However, because juvenile survival was uniformly high (~100%) across populations, variation in fecundity was more likely to explain the observed differences in population fitness.

## Discussion

Using a G x G x G experimental design, this study revealed that DENV blocking by *Wolbachia* in *Ae. aegypti* mosquitoes is influenced by complex interactions between the mosquito’s genetic background, *Wolbachia* strain, and viral serotype. While both *w*AlbB and *w*MelM strains effectively suppressed DENV infection overall, the extent of blocking varied depending on these interacting factors, with *w*MelM conferring stronger and more consistent blocking than *w*AlbB across diverse *Ae. aegypti* populations. This effect was especially pronounced in mosquitoes from Brazil, where *w*MelM showed the greatest reduction in both DENV prevalence and viral load. We also note that the Peruvian population exhibited consistently weaker blocking across strains and serotypes, highlighting a population background in which *Wolbachia*-mediated suppression may be least effective. Blocking was also more effective against DENV-2 than DENV-1 across all *Wolbachia* strains and mosquito populations, suggesting that DENV serotype influences the degree of *Wolbachia*-mediated pathogen blocking. Previous studies have shown that both mosquito genotype and *Wolbachia* strain influence the strength of virus blocking [25]. However, the relative blocking performance of different *Wolbachia* strains appears to be context dependent. For example, in this particular study, stronger inhibition of dengue transmission by *w*AlbB than by *w*MelCS was reported under their experiment, particularly when patient-derived viremic blood meals were used [25]. In our study, by contrast, *w*MelM generally showed stronger blocking than *w*AlbB across several South American mosquito populations. These differences may reflect variation in experimental design, including blood-meal source (patient-derived versus laboratory-spiked), viral isolate, serotype, and titer, mosquito genetic background, and the specific *Wolbachia* variant evaluated. In addition, comparative studies across all four DENV serotypes have found DENV-1 to be more difficult to inhibit than DENV-2 [43], supporting our observation of serotype-dependent variation in blocking. These prior findings align with the trends observed across our South American mosquito populations.

The observed variability in DENV blocking may be driven, in part, by differences in host–symbiont genetic compatibility between *Wolbachia* strains and their mosquito hosts. Genetic compatibility refers to how effectively a *Wolbachia* strain can replicate, localize, and exert pathogen-blocking effects within a given mosquito genotype, shaped by interactions between both the nuclear and mitochondrial genomes. The host cellular environment can support or constrain *Wolbachia* persistence and its ability to modulate host biology in ways that ultimately interfere with DENV replication and dissemination [11,26,44]. Because *Wolbachia* and mitochondria are maternally inherited, introgression and backcrossing strategies used to localize *Wolbachia* strains into field-adapted mosquito populations may generate mismatched nuclear–mitochondrial combinations, potentially affecting symbiont performance and mosquito viability [45–47]. One plausible mechanism underlying these background-dependent fitness effects is cyto-nuclear incompatibility between mosquito nuclear genomes, mitochondrial haplotypes, and *Wolbachia* strains. Publicly available nuclear and mitochondrial genomic datasets from South American *Ae. aegypti* populations could be leveraged in future studies to identify polymorphisms or haplotypes associated with differential *Wolbachia* compatibility. Integrating such genomic information with phenotypic fitness measurements would help clarify the mechanistic basis of host–strain interactions and improve predictions of *Wolbachia* performance in diverse field settings.

Understanding the role of host–symbiont genetic compatibility has important implications for the global scalability of *Wolbachia*-based interventions. Despite being a single global species, given its relatively recent expansion out of the African continent, *Ae. aegypti* populations show substantial genetic differentiation across geographic regions. For example, pairwise F_st_ values are 0.119 for African to South America, and 0.114 for South America to Asian populations [47]. Within the South American continent, the intrapopulation F_st_ value is as high as 0.181 [43]. This underlying genetic structure across the continent likely reflects invasion history and population-specific adaptations to local environments. In terms of the latter, traits such as dengue virus susceptibility [48], thermal tolerance [49], and insecticide resistance [50] are known to vary among *Ae. aegypti* populations due to local selection pressures. As such, the success of *Wolbachia* interventions may depend heavily on how well released strains interact with these locally adapted genetic backgrounds.

Importantly, the variations in these interactions were observed not only in blocking efficacy but also in mosquito fitness. While both *Wolbachia* strains showed a significant fitness cost effect in our experimental settings, variation among host backgrounds suggests fitness outcomes may be background-dependent. For instance, survival outcomes and mosquito development rates differed depending on both the *Wolbachia* strain and the host population. In some populations like Brazil, both *Wolbachia* strains significantly reduced lifespan, with no difference in the scale of that effect between the strains. However, in both Peru and Paraguay, being infected with *w*MelM provided these populations a longer lifespan than those infected with *w*AlbB. These variations can also be seen when we look at the mosquito development rate. Comparisons of just the *Wolbachia*-free populations alone showed differences in their development rate, suggesting that mosquito genetic background on its own influences how quickly these mosquitoes can develop. *Wolbachia* infection, in some cases, can either slow development or, have no effect. The direction and magnitude of change depend on the *Wolbachia* strain and mosquito population. Using a composite fitness model, based on the various fitness parameters that were measured, *w*AlbB-infected mosquitoes had better population fitness relative to both *w*MelM-infected and *Wolbachia*-free mosquitoes. Consistent with previous studies, *w*AlbB infection has been reported to retain several traits favorable for invasion in *Ae. aegypti*, including strong cytoplasmic incompatibility, perfect maternal transmission, and limited effects on some fitness traits [51]. It is important to note that the stage-structured population growth model used here assumes constant laboratory conditions and does not incorporate density dependence or environmental heterogeneity. As a result, estimates of *r*_max_ reflect relative fitness differences under controlled conditions rather than absolute predictions of population growth in the field. In natural settings, factors such as fluctuating temperature, resource limitation, larval competition, and spatial structure are likely to modify both mosquito demography and *Wolbachia* dynamics. Despite these limitations, laboratory-based estimates of population growth remain valuable for comparative assessments of fitness trade-offs among treatments and provide a useful baseline for informing more complex, field-relevant models.

The results from our study align well with previous work that have shown that mosquito and *Wolbachia* genetic background play an important role in mosquito fitness. For instance, one study that looked at the fecundity of both *w*AlbB and *w*MelM-infected *Ae. aegypti* (Brazilian genetic background) found that *w*MelM-infected mosquitoes laid significantly fewer eggs than *w*AlbB-infected mosquitoes [30]. Notably our findings may help explain field observations in countries like Brazil (Jurujuba) [52] and Colombia (Medellín) [18] where, *w*Mel *Wolbachia* invasion has been slower or less stable than expected. In particular, the fitness costs we observed in Brazilian (Rio de Janeiro) populations infected with either *Wolbachia* strain could contribute to reduced establishment and spread in the field. In this context, the reduced population fitness observed for wMelM in the Brazilian genetic background provides a plausible explanation for the slower or less stable invasion dynamics. If similar host-*Wolbachia* compatibility effects occur in the field, even modest fitness costs could limit the rate of spread or long-term persistence of specific *Wolbachia* strains, despite strong virus-blocking efficacy.

The presence of *Wolbachia* may alter key metabolic and physiological processes in *Ae. aegypti*, leading to trade-offs that impact host fitness, although the magnitude of these effects may vary across mosquito populations. These alterations include increased energetic demands to support symbiont replication and maintenance, competition for essential cellular resources, and modulation of host immune and stress response pathways [9,12,53]. For instance, *Wolbachia* can influence lipid metabolism and reactive oxygen species levels, potentially diverting energy away from other vital functions such as reproduction and longevity [10,54]. These physiological shifts might reduce host fitness in a context-dependent manner, as evidenced by variation in survival across mosquito genetic backgrounds in our study.

Together, these findings support the idea that genetic interactions between host, symbiont, and virus shape the outcomes of *Wolbachia*-mediated pathogen blocking, invasiveness and ultimately the epidemiological outcome of *Wolbachia* population replacement as a dengue mitigation strategy. Overall, our study has shown *w*MelM blocked DENV more effectively than *w*AlbB, but the strength of blocking varied depending on the mosquito population and DENV serotype. The Brazilian population tended to exhibit the strongest blocking, and the Peruvian, the worst. In terms of fitness, *w*AlbB-infected mosquitoes consistently showed higher fitness compared to *w*MelM, but fitness effects were not uniform — they depended on the mosquito population and fitness trait measured. To further investigate the mechanisms underlying these interactions, future studies could incorporate genomic and transcriptomic analyses. Sequencing the genomes of the mosquito populations used in this study could identify key variants that mediate *Wolbachia* compatibility or viral susceptibility. In parallel, transcriptomic profiling could reveal how gene expression patterns shift across host-symbiont-virus combinations, offering insights into host pathways that regulate blocking or fitness outcomes. Ultimately, such mechanistic work will be valuable for complementing phenotypic studies and refining deployment strategies. In particular, genotype-specific differences in virus blocking and fitness could be used to inform strain-matching approaches in which *Wolbachia* strains are selected based on their compatibility with local mosquito genetic backgrounds to balance virus suppression and population persistence. These interaction effects could also be incorporated into predictive models of *Wolbachia* invasion and impact, improving forecasts of establishment success and long-term efficacy across tropical regions. Ideally, *Wolbachia* interventions could be tailored to specific ecological and genetic contexts—matching mosquito strains with compatible *Wolbachia* infections to maximize blocking while minimizing fitness costs in target environments. However, achieving that level of precision requires significant time, resources, and localized data. Given that *Wolbachia* deployment and introgression can take several months, it may not be suitable as a rapid response during active dengue outbreaks. Instead, these efforts could be viewed as part of long-term, regionally informed preparedness strategies aimed at reducing the risk and severity of future outbreaks.

## Supporting information

S1 Fig Dengue prevalence in the carcass of mosquito populations from Brazil, Paraguay, and Peru tested with *Wolbachia* strains *w*AlbB, *w*MelM, and *Wolbachia* -free controls. Each population was exposed to a) DENV-1 or b) DENV-2, and mosquitoes were subsequently collected at 5-, 10-, and 15-days post-infection (DPI) to assess dengue prevalence in the carcasses. N = 12 individuals per treatment.
(TIF)

S2 Fig Dengue viral load in the carcass of mosquito populations from Brazil, Paraguay, and Peru tested with *Wolbachia* strains *w*AlbB, *w*MelM, and *Wolbachia* - free controls. Each population was exposed to DENV-1 or DENV-2, and dengue viral loads were assessed in carcasses collected at 5-, 10-, and 15- days post-infection (DPI). Bars represent median viral load and whiskers indicating the 95% confidence intervals. Each dot represents carcass tissue dissected from a single individual. N = 12 individuals per treatment.
(TIF)

S1 Table Results of binomial generalized linear models (GLMs) testing the effects of mosquito population (country), *Wolbachia* infection status, dengue virus (DENV) serotype, and days post-infection (DPI), including all interaction terms, on dengue infection prevalence in *Aedes aegypti.* Infection prevalence was modeled as the number of infected individuals out of the total sampled per treatment group. Statistical significance was assessed using likelihood ratio tests.
(DOCX)

S2 Table Summary of ANOVA results from mixed-effects models testing the effects of mosquito population (country), *Wolbachia* infection status, dengue virus (DENV) serotype, and days post-infection (DPI), as well as their interactions, on dengue viral load in *Ae. aegypti.* (DOCX)

S1 Data 5. Full pairwise comparisons of dengue viral load across mosquito populations, *Wolbachia* infection states, dengue virus (DENV) serotypes, and days post-infection (DPI). Pairwise differences, standard errors, test statistics, and adjusted p-values are reported for all comparisons.
(XLSX)

S3 Table Summary of ANOVA results testing the effects of *Wolbachia* strain, mosquito population, and gonotrophic cycle, as well as their interactions, on fecundity. (DOCX)

S4 Table Log-rank p-values from pairwise comparison analysis of survival in South American *Ae. aegypti* populations with three *Wolbachia* infectious status- *w*AlbB, *w*MelM, and *Wolbachia* -free controls. (DOCX)

S5 Table Fitness (*r*_max_) values derived from life-history traits measured in mosquitoes reared from three populations (Brazil, Paraguay, Peru) and three *Wolbachia* infection statuses (*Wolbachia*-free, *w*MelM, *w*AlbB). The 95% credible intervals are derived from the posterior distribution.
(DOCX)
