# Supplementary material for: Interactions between mosquito genetic background and Wolbachia strain affect dengue virus blocking and fitness in South American populations of Aedes aegypti
Source: PLoS Negl Trop Dis. 2026 May 27;20(5):e0014403. doi: 10.1371/journal.pntd.0014403 (PMC13245867; doi:10.1371/journal.pntd.0014403)

S1 Table. Results of binomial generalized linear models (GLMs) testing the effects of mosquito population (country), *Wolbachia* infection status, dengue virus (DENV) serotype, and days post-infection (DPI), including all interaction terms, on dengue infection prevalence in Aedes aegypti. Infection prevalence was modeled as the number of infected individuals out of the total sampled per treatment group. Statistical significance was assessed using likelihood ratio tests.


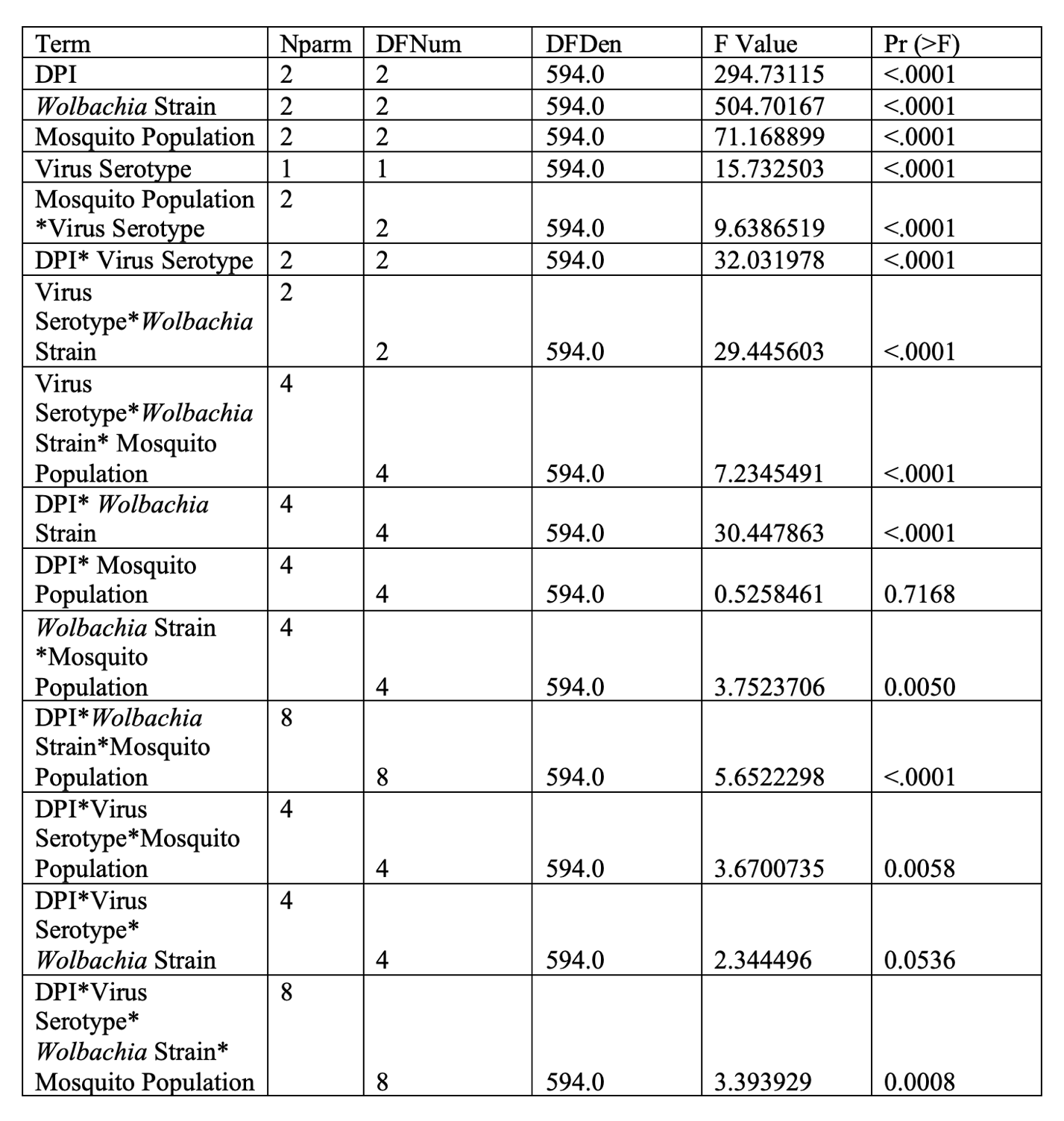

Supplement: S1 Table — Infection prevalence was modeled as the number of infected individuals out of the total sampled per treatment group. Statistical significance was assessed using likelihood ratio tests. (DOCX) [file pntd.0014403.s003.docx]
