# Supplementary material for: Interactions between mosquito genetic background and Wolbachia strain affect dengue virus blocking and fitness in South American populations of Aedes aegypti
Source: PLoS Negl Trop Dis. 2026 May 27;20(5):e0014403. doi: 10.1371/journal.pntd.0014403 (PMC13245867; doi:10.1371/journal.pntd.0014403)

S2 Table. Summary of ANOVA results from mixed-effects models testing the effects of mosquito population (country), *Wolbachia* infection status, dengue virus (DENV) serotype, and days post-infection (DPI), as well as their interactions, on dengue viral load in Ae. aegypti.


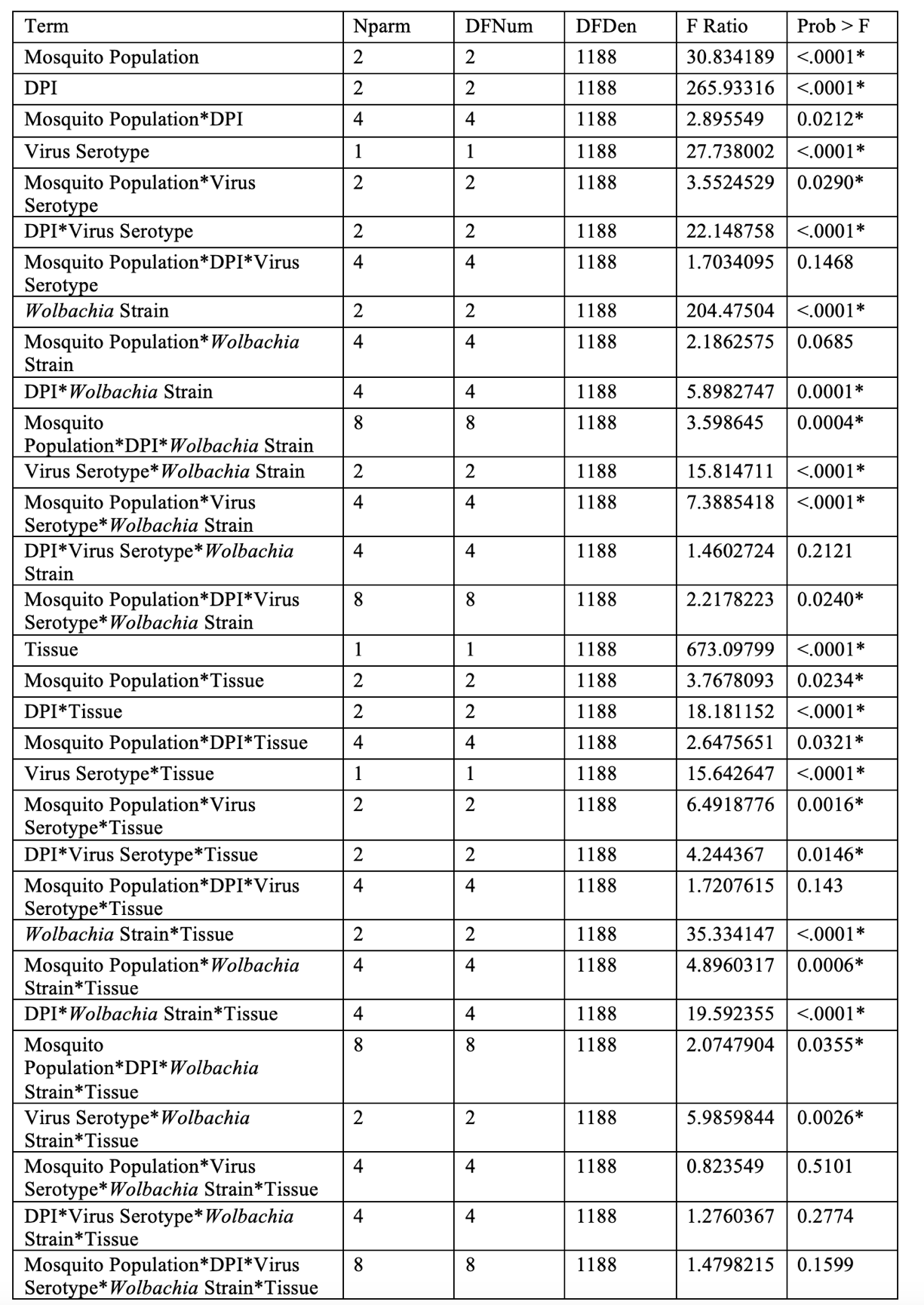

Supplement: S2 Table — (DOCX) [file pntd.0014403.s004.docx]
