# Supplementary material for: Interactions between mosquito genetic background and Wolbachia strain affect dengue virus blocking and fitness in South American populations of Aedes aegypti
Source: PLoS Negl Trop Dis. 2026 May 27;20(5):e0014403. doi: 10.1371/journal.pntd.0014403 (PMC13245867; doi:10.1371/journal.pntd.0014403)

S6 Table. Summary of ANOVA results testing the effects of *Wolbachia* strain, mosquito population, and gonotrophic cycle, as well as their interactions, on fecundity.


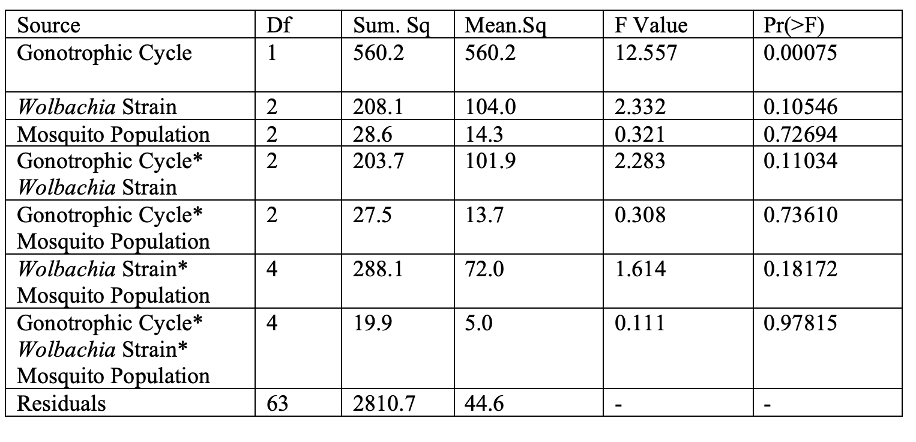

Supplement: S3 Table — (DOCX) [file pntd.0014403.s006.docx]
