# Supplementary material for: Interactions between mosquito genetic background and Wolbachia strain affect dengue virus blocking and fitness in South American populations of Aedes aegypti
Source: PLoS Negl Trop Dis. 2026 May 27;20(5):e0014403. doi: 10.1371/journal.pntd.0014403 (PMC13245867; doi:10.1371/journal.pntd.0014403)

S7 Table. Log-rank p-values from pairwise comparison analysis of survival in South American *Ae. aegypti* populations with three *Wolbachia* infectious status- *w*AlbB,

*w*MelM, and *Wolbachia* -free controls.


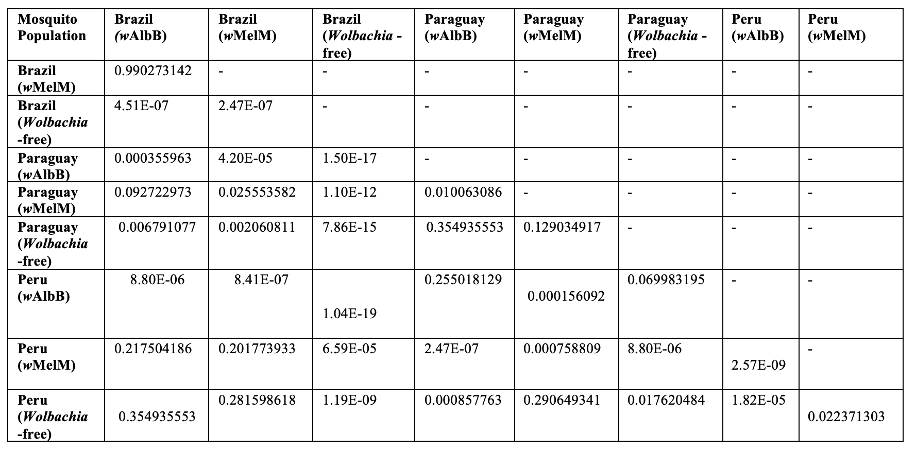

Supplement: S4 Table — (DOCX) [file pntd.0014403.s007.docx]
