# Supplementary material for: Interactions between mosquito genetic background and Wolbachia strain affect dengue virus blocking and fitness in South American populations of Aedes aegypti
Source: PLoS Negl Trop Dis. 2026 May 27;20(5):e0014403. doi: 10.1371/journal.pntd.0014403 (PMC13245867; doi:10.1371/journal.pntd.0014403)
Supplement: S5 Table — The 95% credible intervals are derived from the posterior distribution. (DOCX) [file pntd.0014403.s008.docx]

S3 Table. Fitness (*r*_max_) values derived from life-history traits measured in mosquitoes reared from three populations (Brazil, Paraguay, Peru) and three *Wolbachia* infection statuses (*Wolbachia*-free, *w*MelM, *w*AlbB). The 95% credible intervals are derived from the posterior distribution.

**
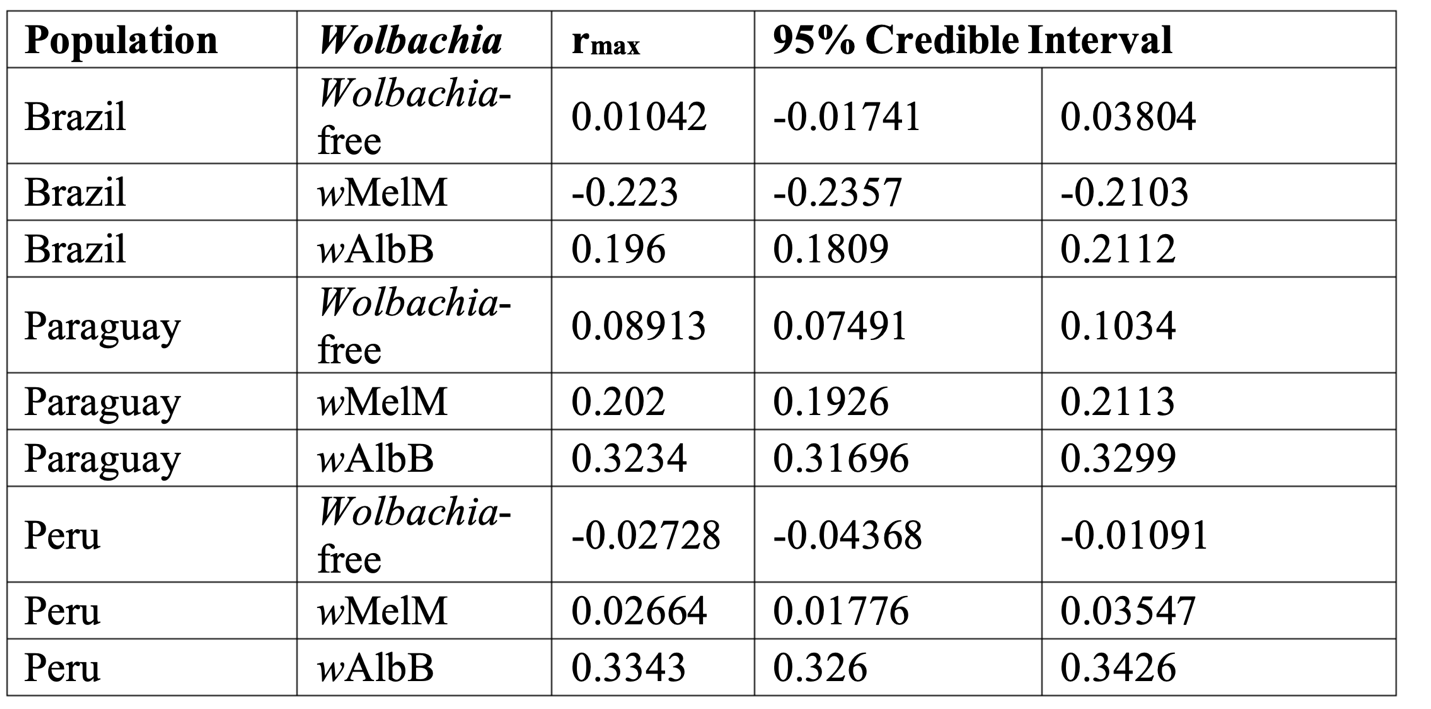
**
